# A Rare Case of Necrotising Fasciitis of the Breast Arising From Undiagnosed Breast Cancer

**DOI:** 10.7759/cureus.104977

**Published:** 2026-03-10

**Authors:** Tamara Fisher, April Miu

**Affiliations:** 1 Department of General Surgery, Townsville University Hospital, Townsville, AUS

**Keywords:** breast cancer, breast disease, emergency mastectomy, necrotising fasciitis (nf), polymicrobial

## Abstract

Necrotising fasciitis is a rapidly progressing soft tissue infection that rarely occurs in the breast. Clinical features include erythema, swelling, skin necrosis and bullae, with pain disproportionate to examination. Early recognition coupled with prompt initiation of broad-spectrum antibiotics and surgical debridement is paramount in preventing multiorgan dysfunction and mortality. We describe a 61-year-old woman with a rare case of necrotising fasciitis of the breast arising from undiagnosed underlying breast cancer. This patient underwent urgent mastectomy for source control, supplemented by broad-spectrum antimicrobial therapy.

## Introduction

Necrotising fasciitis is a life-threatening soft tissue infection most commonly affecting the limbs, perineum and abdominal wall [1]. It is characterised by progressive necrosis of the muscle fascia and overlying subcutaneous fat and skin. Muscle is often spared due to its generous blood supply. Conversely, the relatively poor blood supply of the fascia results in rapid spread of infection along the fascial plane, resulting in tissue ischaemia, necrosis and systemic toxicity [2,3]. Clinical features include rapid progression of erythema and oedema with severe pain out of proportion to examination findings. Other cutaneous signs include skin bullae, necrosis and crepitus. Systemic signs such as fever and haemodynamic instability are common, with a high risk of progression to multiorgan failure and death if not identified and treated in a timely manner [4]. Treatment of necrotising fasciitis involves prompt fluid resuscitation and administration of broad-spectrum intravenous antibiotics, alongside early surgical referral for urgent debridement of necrotic and nonviable tissue [2].

Necrotising fasciitis of the breast is extremely rare, with only a limited number of cases reported in the literature. It most commonly occurs after an inciting event, such as a traumatic injury, insect bite or after an injection or procedure. Conversely, primary necrotising fasciitis refers to primary infection arising without an inciting event. Risk factors include smoking, diabetes, hypertension, obesity and immunosuppression [2,5,6].

We present a 61-year-old woman with a rare case of necrotising fasciitis of the breast arising from underlying breast cancer. Her treatment comprised emergent mastectomy for source control alongside antimicrobial therapy.

## Case presentation

A 61-year-old woman presented to a rural hospital with a two-day history of pain, swelling and malodourous discharge from her right breast. These symptoms were precipitated by a trauma, whereby her breast was accidentally struck by a young relative. This was on a background of having an abnormal lump in her right breast for seven years, which had never been investigated. The patient rarely engaged in healthcare and had no previously diagnosed medical comorbidities, nor did she take any regular medications. She was found to be obese and diagnosed with hypertension during her admission.

**Figure 1:**
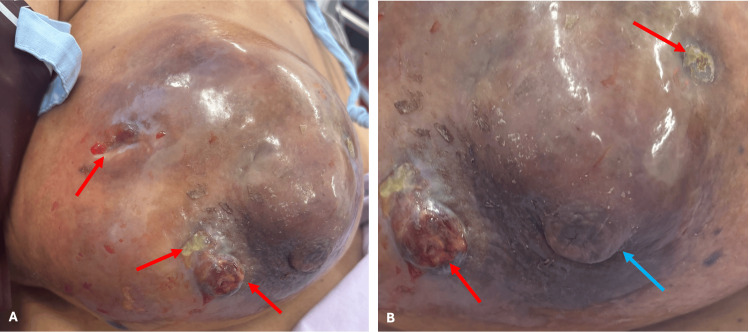
Clinical photos showing a grossly oedematous breast with multiple skin ulcerations (red arrows) with active leakage of fluid, and inferior pointing of the nipple (blue arrow)

**Figure 2:**
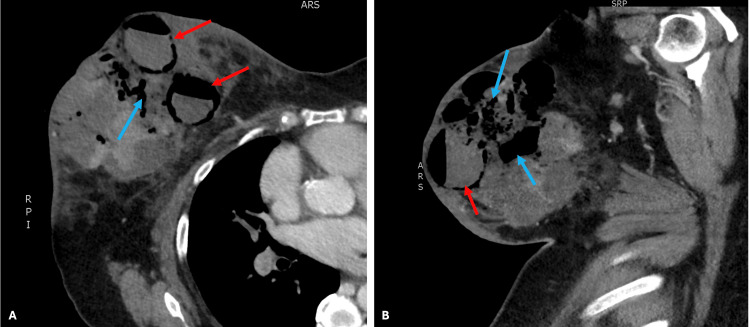
Computed tomography images of the chest showing the right breast: note is made of multiple gas- and fluid-filled collections (red arrows), and subcutaneous gas (blue arrows) A: axial section, B: sagittal section

In the emergency department, she became tachypnoeic, febrile, tachycardic and hypotensive. Examination showed a large oedematous right breast with ulcerative skin changes, discharging dishwater fluid and bubbling gas, with inferior pointing of the nipple (Figure 1). There were palpable crepitations and axillary lymphadenopathy. The left breast was examined normally. Blood showed lactic acidosis and neutrophilia consistent with septic shock (Table 1). A computed tomography (CT) of the chest was performed and revealed a multiloculated breast mass with multiple fluid- and gas-containing collections, as well as free subcutaneous gas (Figure 2). These biochemical and radiological findings supported the clinical diagnosis of necrotising fasciitis.

**Table 1:** Haematological and biochemical investigations at presentation BE: base excess, CRP: C-reactive protein, Hb: haemoglobin, pCO2: partial pressure of carbon dioxide, pH: potential of hydrogen, WCC: white cell count

Investigation	Reference range	Patient’s results
Hb (g/L)	115-160	73
WCC (×10^9^/L)	4.0-11.0	12.9
pH	7.32-7.43	7.20
pCO_2_ (mmHg)	38-54	43
Bicarbonate (mmol/L)	22-33	17
BE (mmol/L)	-2.0-3.0	-10.5
Lactate (mmol/L)	0.5-2.2	9.4
CRP (mg/L)	<5	192

**Figure 3:**
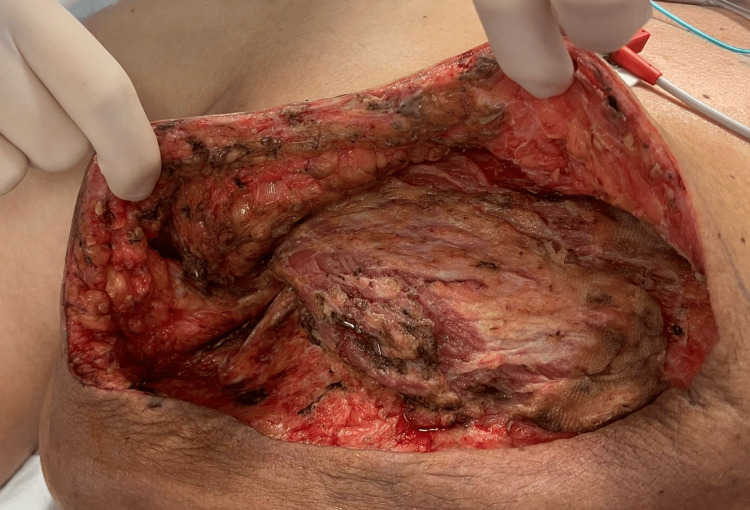
Clinical photo of the wound post-initial debridement

The patient was commenced immediately on broad-spectrum intravenous antibiotics consisting of meropenem 1 g eight-hourly, clindamycin 600 mg eight-hourly, and vancomycin 1.5 g 12-hourly. She then proceeded to emergent debridement in the operating theatre by way of a mastectomy. The excised tissue was sent for both culture and histopathology. The skin flaps were left open, and the wound packed with betadine-soaked gauze. The patient was then urgently transferred to a tertiary facility for ongoing intensive care. She proceeded to a planned re-look operation 48 hours after the initial debridement. The wound appeared to have a clean and healthy base, and no further tissue necrosis was found (Figure 3). The wound was then closed in layers and dressed with negative-pressure wound therapy.

The tissue and fluid cultures from the initial debridement grew *Streptococcus agalactiae*, *Klebsiella pneumoniae* and *Clostridium septicum*. Initial blood cultures were also positive for *Clostridium septicum*. Her antibiotic therapy was accordingly rationalised on day 3 post-presentation to intravenous piperacillin/tazobactam 4 g/0.5 g eight-hourly. After seven days of piperacillin/tazobactam, her antibiotic therapy was de-escalated to oral amoxicillin/clavulanic acid 875 mg/125 mg twice daily for a further two weeks.

The patient unfortunately developed multiple complications within her first week of hospitalisation. She suffered a type II myocardial infarction with a troponin rise to 7,464 ng/L (reference range: <34 ng/L). Fortunately, an echocardiogram excluded any significant ventricular or valvular pathologies. She also developed bilateral pulmonary embolisms without evidence of right heart strain and was commenced on a heparin infusion, which was switched to therapeutic enoxaparin 24 hours later. She subsequently developed a spontaneous haematoma at the mastectomy site, which required multiple blood transfusions and urgent evacuation in theatre owing to haemodynamic instability. No further wound complications were observed after this event. She made a gradual recovery and was discharged home 21 days after her initial presentation.

Her histology was eventually reported as 140 mm of grade 2 invasive carcinoma of no special type with involved anterior and lateral margins. There was also evidence of likely pulmonary metastases seen on CT of the chest. The patient was commenced on endocrine therapy per the consensus of the multidisciplinary team meeting, with a plan to follow up with medical oncology in the outpatient setting to discuss systemic therapy.

## Discussion

Necrotising fasciitis is a rapidly spreading soft tissue infection of the fascia and subcutaneous tissue [3]. Necrotising fasciitis of the breast is a rare condition, largely reported in the literature by way of case studies. It most often occurs secondary to a traumatic event such as direct injury or insect bite, or medical procedures such as needle aspiration, core biopsy or surgery [6]. The presented patient had a minor trauma to the breast as an inciting incident on a background of an undiagnosed breast cancer, and her risk factors included obesity and hypertension.

Prompt diagnosis is imperative both for breast salvage and survival. Necrotising fasciitis of the breast can be difficult to diagnose clinically as cutaneous findings can be less apparent owing to the thickened tissue between the deep fascia and skin [2]. Most commonly, a mastectomy is required to achieve adequate source control, but there have been some reported cases of serial debridements and partial mastectomy where tissue necrosis was less extensive [7].

While the diagnosis of necrotising fasciitis is made clinically, pathology and imaging can assist in diagnosis and prognosis, although they should not delay surgical management [8]. The CT scan performed for this patient demonstrated extensive subcutaneous gas with multiple fluid-filled collections, consistent radiologically with necrotising fasciitis.

The first reported case of breast necrotising fasciitis was by Shah et al. in 2001 [9]. They describe a six-step management plan that has been echoed in successive case studies in the literature. The plan highlights the importance of early recognition and surgical consultation, coupled with prompt resuscitation with intravenous fluids and administration of broad-spectrum antibiotics. They advocate for diagnostic incision into the affected site to assess the underlying fascia, followed by radical excision of the ‘pseudotumour’, that being all necrotic and nonviable tissue. Pus should be sampled and sent for urgent gram stain and culture to allow for targeted antimicrobial therapy. The wound should be inspected under anaesthesia to confirm adequacy of the excision at 24 hours. Only once devitalised tissue has been adequately cleared should reconstruction commence. They suggest delaying reconstruction by several months.

It must be clarified that while some cases of necrotising fasciitis of the breast require mastectomy for source control, serial debridement and partial mastectomy have allowed for successful surgical treatment in many reported cases. The principles of surgical management are to remove all necrotic and nonviable tissue while conserving as much healthy breast tissue as possible [2]. Unfortunately, in this instance, mastectomy was required to obtain complete infection control, as demonstrated by the depth of involved tissue observed on cross-sectional imaging (Figure 2). The recommendation by Shah et al. to delay reconstruction is also heavily contended in the literature. There are many reported cases of reconstruction occurring as early as the second-look operation. In recent publications, reconstruction typically occurs within two weeks of the final source control operation. Split-thickness skin grafting is the main method of reconstruction, as opposed to delayed primary closure as in the presented case [2].

Necrotising fasciitis is classified into two microbiologic categories. Type I ‘polymicrobial’ necrotising infection is caused by mixed aerobic and anaerobic bacteria. Type II ‘monomicrobial’ necrotising infection is caused by a single organism, most commonly group A *Streptococcus* or *Staphylococcus aureus* [3]. This patient had type I necrotising fasciitis, which is the more common of the two subtypes, hence the need for broad-spectrum empirical antibiotic coverage [7].

There have been only two other documented cases of necrotising infection associated with underlying malignancy. One in a 42-year-old patient who underwent localised debridement only and an interval modified radical mastectomy [10], and the other in a 56-year-old patient who underwent a modified radical mastectomy for source control [1]. Both received broad-spectrum antibiotic treatment.

We describe a rare case of breast necrotising fasciitis following a minor trauma to the breast, on a background of undiagnosed underlying breast cancer. She was managed appropriately with prompt fluid resuscitation, broad-spectrum intravenous antibiotics and early surgical debridement. Early primary closure of the wound was able to be achieved, given the extensive initial debridement. The haematoma that developed in this patient while she was newly therapeutically anticoagulated was an unfortunate complication. Nonetheless, the wound was able to be closed again primarily after the haematoma was evacuated, with no further wound complications arising.

## Conclusions

Necrotising fasciitis of the breast is a rare condition. In this case, necrotising fasciitis was suspected on clinical grounds, and the diagnosis was supported by laboratory findings and cross-sectional imaging, and ultimately confirmed by diagnostic incision. This case highlights the importance of having a high index of suspicion for necrotising infection and ensuring early surgical referral. It is imperative that once suspected, the patient is treated with broad-spectrum antibiotics, given that most necrotising infections are polymicrobial. Prompt surgical debridement of all necrotic tissue is paramount, and a staged re-look debridement in theatre within 24-48 hours is recommended to ensure no further necrotic or nonviable tissue remains. Reconstruction should be individualised. In this case, primary closure was achieved at the second operation due to successful debridement at the index operation.
